# Complementary and alternative medicine use among elderly cancer patients: a cross-sectional study in South Korea

**DOI:** 10.3389/fonc.2026.1762467

**Published:** 2026-04-15

**Authors:** Hyunyem Chang, Soo Jeung Choi, Hyea Bin Im, Dain Choi, Dongwoon Han

**Affiliations:** 1 Veterans Hospital System Medical Center, Seoul, Republic of Korea; 2 Department of Global Health and Development, Graduate School, Hanyang University, Seoul, Republic of Korea; 3 Institute of Health Services Management, Hanyang University, Seoul, Republic of Korea; 4 Department of Preventive Medicine, Hanyang University College of Medicine, Seoul, Republic of Korea

**Keywords:** aged, complementary therapies, cross-sectional studies, neoplasms, Republic of Korea

## Abstract

**Introduction:**

The use of complementary and alternative medicine (CAM) is common among elderly male cancer patients; however, evidence is limited on its prevalence, characteristics, and determinants in Korea. This study examined the prevalence, patterns, and reasons for CAM use, and identified factors associated with its use among elderly male cancer survivors.

**Materials and methods:**

A cross-sectional survey was conducted with 420 cancer patients aged ≥65 years attending outpatient clinics of the Veterans Hospital System, a 1,000-bed secondary hospital in Seoul, Korea. Data on CAM utilization, modalities, reasons for use, and information sources were collected using structured questionnaires. Multivariate logistic regression was used to identify demographic and clinical factors associated with CAM use.

**Results:**

Among participants, 60.0% reported using CAM. The most common modalities were exercise (brisk walking, 49.4%) and dietary interventions (42.9%). The primary reason for use was immune enhancement (61.6%), and family members or relatives were the main information source (42.1%). Multivariate analysis revealed that being married (OR 2.49, 95% CI 1.40–4.45), having prostate cancer (OR 2.14, 95% CI 1.36–3.35), and undergoing surgery (OR 1.62, 95% CI 1.07–2.45) were significantly associated with CAM use.

**Conclusion:**

CAM use is highly prevalent among elderly male Korean cancer patients, particularly among married men, prostate cancer patients, and those who have undergone surgery. Oncologists should incorporate discussions about CAM into survivorship care, and further studies are warranted to assess the impact of CAM modalities on quality of life and clinical outcomes.

## 1 Introduction

The global aging trend is accelerating at an unprecedented pace, presenting formidable challenges to healthcare systems worldwide. According to the World Health Organization, the elderly population is projected to rise dramatically, reaching 2.1 billion by 2050 (1). This demographic shift is particularly pronounced in countries like Japan and Korea, where the elderly are expected to comprise 38% and 46.4% of the population by 2070, respectively, profoundly impacting their healthcare systems (2). Such shifts significantly strain healthcare resources and amplify the burden of diseases like cancer, which is increasingly prevalent among the elderly (3). Elderly cancer patients face compounded challenges due to their heightened vulnerability to treatment-related side effects (4),. In Europe, the widespread use of dietary supplements among over half of cancer patients necessitates proactive intervention by healthcare providers (5). In Korea, lifestyle factors such as smoking and poor dietary habits place elderly men at greater risk, underscoring the urgency for targeted investigations into self-management (6).

Elderly cancer patients have complex needs, often intertwined with mental health problems, as assessed by the Hospital Anxiety and Depression Scale (HADS) (7). Vitamins, ginseng, and green tea are widely used as complementary approaches for managing specific health conditions such as immune dysfunction, cancer, high cholesterol, and digestive disorders. Ginseng and green tea have also been utilized as supportive interventions for heart disease, anxiety, and depression (8). Yoga has been recognized for its value in reducing stress, enhancing immune function, and improving flexibility, muscle strength, and balance. It has been employed as a form of CAM that offers therapeutic benefits to patients undergoing cancer treatment (9).

Given these complexities, integrating CAM into supportive care for elderly cancer patients merits greater attention. Evidence suggests that those who engage in CAM practices, including spiritual practices such as personal prayer and meditation, report improved psychological wellbeing and quality of life (10). Research indicates that personalized CAM interventions, aligned with patients’ cultural and individual preferences, may lead to better adherence to treatment plans and help mitigate anxiety and depression (11). This underscores the need for a holistic, patient-centered approach that respects the diverse practices and beliefs of elderly cancer patients.

Despite these potential benefits, supportive care incorporating CAM for elderly cancer patients remains limited. The integration of complementary medicine into conventional cancer care is still not widespread, complicating medical decision-making. Geographic and cultural contexts significantly influence CAM utilization patterns among cancer patients (12). While certain CAM practices are prevalent in specific regions, such as mushroom usage in Japan and traditional Chinese medicine in China, and dietary interventions and acupuncture in Taiwan among (13, 14), comprehensive studies focusing on CAM use among elderly cancer patients in major Asian nations, and specifically in Korea, remain scarce. Most previous surveys have primarily described the prevalence or patterns of CAM use, with less attention paid to how social support, treatment trajectory, and emotional vulnerability may jointly influence CAM-related health-seeking behavior in this population (15, 16). In elderly cancer patients, marital status may reflect the availability of social support and shared decision-making, while cancer type and treatment modality may shape symptom burden and perceived supportive care needs. Psychological factors such as anxiety and depression may further influence motivation to seek self-management strategies, including CAM (17). Therefore, the objectives of this study were (1) to investigate the patterns and characteristics of CAM use among elderly male cancer patients in Korea and (2) to identify demographic, clinical, and psychological factors associated with CAM utilization in this population.

## 2 Materials and methods

### 2.1 Setting and design

This study was conducted at the Veterans Hospital System (VHS) in Seoul, South Korea, targeting male outpatients aged 65 years and older. A convenience sampling method was used, and cancer patients receiving care in six outpatient departments were surveyed: hematology–oncology, surgery, day wards, radiation oncology, gastroenterology, and urology. During the recruitment period, 600 eligible patients were identified, and 450 were invited to participate via an invitation letter and a follow-up phone call. Of these, 420 agreed to participate (participation rate: 93.3%), while 30 declined. After excluding 12 incomplete questionnaires, data from 408 participants were included in the final analysis (response rate: 97.1%). The data were collected from cancer patients attending six outpatient clinics between 25 August and 15 September 2021. Face-to-face interviews were conducted by trained interviewers, each lasting approximately 10–15 min. Each participant who completed the survey was provided with a KRW 5,000 gift voucher as a token of appreciation. Because participants were recruited using a convenience sampling method in an outpatient setting and data were collected through interview-based self-report questionnaires, the study was potentially subject to selection bias, recall bias, and social desirability bias.

### 2.2 Study population and survey administration

Eligible participants were cancer patients aged 65 years or older who had been diagnosed within the past 5 years, were able to understand and respond to interview questions, and voluntarily agreed to participate. Patients with physical or cognitive impairments that hindered completion of the questionnaire were excluded. Using a convenience sampling strategy, participants were recruited consecutively in the outpatient setting until the target sample size was reached within the study timeframe. Given the characteristics of the study setting, the final sample consisted entirely of older male veterans receiving outpatient cancer care. The sample size for this study was calculated using the formula below, based on a 45.7% CAM utilization rate among elderly cancer patients (the average utilization rate derived from existing CAM utilization studies). The minimum required sample size was calculated as 381 participants. Considering a 10% reduction in response rate, the goal was to recruit a total of 420 subjects.

**Table:** .

$n\;=\;z_{a/2}^{2}\cdot \frac{pq}{d^{2}}$	p= estimated proportion of CAM use from previous studies
	q = the proportion of elderly cancer patients not using CAM (1-p)
	Z_0.025_ = 1.96, 95% confidence interval (CI)
n= sample size	d= 0.05, the margin of error

### 2.3 Dependent variables

The final questionnaire comprised four sections with a total of 34 items. To ensure clarity and consistency, all participants were presented with the World Health Organization’s definition of complementary and alternative medicine (CAM) before being asked about their CAM use. Respondents were then asked about their CAM utilization and the specific types of CAM used, which were categorized into five major groups. CAM was also classified according to the National Center for Complementary and Integrative Health (NCCIH) framework, which included lifestyle-based practices such as brisk walking under movement therapy. Additional questions explored reasons for CAM use, consultation with a physician regarding CAM, methods of accessing CAM, and associated out-of-pocket costs. For non-users, reasons for not using CAM were recorded. Finally, participants’ CAM-related practices were evaluated (18, 19).

### 2.4 Independent variables

The questionnaire assessed patients’ health status and disease characteristics, including relevant clinical features. Depression and anxiety were measured using the Hospital Anxiety and Depression Scale (HADS), following the guidelines of the National Comprehensive Cancer Network (NCCN). Previous studies have shown that the HADS has good internal consistency (Cronbach’s α = 0.80–0.90) (7, 20). There were no missing data in this study. Stress and pain were measured using the Korean version of the pain assessment tool and problem list developed in accordance with the NCCN guidelines (February 2021 version). A cutoff score of ≥4 was used to indicate clinically significant distress, according to the NCCN guidelines and previous validation studies of the Korean version (21). Additional items were used to collect information about the cancer treatment process, including diagnosis, staging, and treatment methods, as well as participants’ experiences with healthcare services.

### 2.5 Covariates

The first section of the questionnaire collected demographic and socioeconomic characteristics, including age, education level, marital status (married, divorced, widowed), and monthly household income (18).

### 2.6 Statistical analysis

All analyses were conducted using the Statistical Package for the Social Sciences (SPSS) version 26.0 (IBM Corp., Armonk, NY, USA). Descriptive statistics were used to summarize the sociodemographic and clinical characteristics of the study population. Differences between CAM users and non-users were assessed using the chi-square test. Candidate variables considered for regression analysis included sociodemographic, clinical, and psychological factors assessed in the survey. Variables showing statistical significance in univariate analysis were subsequently entered into the multivariable logistic regression model to identify factors independently associated with CAM use. Odds ratios (ORs) and 95% confidence intervals (CIs) were calculated. Statistical significance was set at *p* < 0.05.

## 3 Results

### 3.1 Characteristics of the participants

The sociodemographic and clinical characteristics of the 408 participants are presented in **Table 1**. Nearly half of participants were 75 to 79 years of age (45.8%). Most participants had attained a high school level of education (39.9%) and were married (79.9%). A statistically significant association was identified between marital status and CAM utilization, with CAM use being more common among those with a spouse (p = 0.002).

**Table 1:** Sociodemographic and clinical characteristics of the participants (n = 408).

Variables	Total N (%)	Users (N = 245)	Non-users (N = 163)	*P-*value
Gender				
Male	408 (100%)	245 (60.0)	163 (40.0)	
Female	0			
Age				
Mean ± SD	75.85 ± 5.87	75.96 ± 5.40	75.74 ± 6.34	
65–74	161 (39.4)	95 (38.8)	66 (40.4)	0.717
75–79	187 (45.8)	116 (47.3)	71 (43.6)	
≥43	60 (14.8)	34 (13.9)	26 (16.0)	
Educational level				
Below middle school	155 (38.0)	92 (37.6)	63 (38.7)	0.752
High school	163 (39.9)	99 (40.4)	67 (41.1)	
College level and above	87 (22.1)	54 (22.0)	33 (20.2)	
Marital status				
Spouse	326 (79.9)	184 (75.1)	142 (87.1)	0.002^**^
No spouse ^a^	82 (20.1)	61 (24.9)	21 (12.9)	
Monthly income (KRW)				
≤KRW)lyona	126 (30.8)	69 (28.1)	57 (35.0)	0.752
1,000,000-3,000,000	225 (55.1)	143 (58.3)	82 (50.3)	
≥50.3)000-	57 (14.1)	33 (13.6)	24 (14.7)	
Perceived health status				
Good	203 (49.7)	121 (49.4)	82 (50.3)	0.856
Bad	205 (50.3)	124 (50.6)	81 (49.7)	
Type of cancer^b^ (N = 445)				
Prostate cancer	183 (41.1)	125 (51.0)	58 (35.6)	0.001^**^
Lung cancer	69 (15.5)	35 (14.3)	34 (20.9)	0.056
Rectal, colon cancer	40 (8.9)	22 (9.0)	18 (11.0)	0.301
Stomach cancer	33 (7.4)	18 (7.3)	15 (9.2)	0.310
Liver cancer	24 (5.3)	15 (6.1)	9 (5.5)	0.490
Thyroid cancer	3 (1.0)	1 (0.4)	2 (1.2)	0.351
Others	93 (20.8)	53 (21.6)	40 (24.5)	0.285
Type of treatment ^c^				
Surgery	214 (52.5)	139 (56.7)	75 (46.0)	0.043^*^
Chemotherapy	189 (46.3)	111 (45.3)	78 (47.9)	0.685
Radiotherapy	143 (35.0)	81 (33.1)	62 (38.0)	0.341
Hormone therapy	48 (11.8)	32 (13.1)	16 (9.8)	0.350
Others	44 (10.8)	30 (12.2)	14 (8.6)	0.259
Perceived anxiety level ^d^				
0–7 points	267 (65.4)	169 (68.9)	98 (60.1)	0.016*
8–21 points	141 (34.6)	76 (31.1)	65 (39.9)	
Perceived depression level ^d^				
0–7 points	105 (25.7)	73 (29.8)	32 (19.6)	0.014*
8–21 points	303 (74.3)	172 (70.2)	131 (80.4)	
Perceived distress thermometer level^e^				
< 4 points	159 (39.0)	89 (36.3)	70 (42.9)	0.108
≥ 4 points	249 (61.0)	156 (63.7)	93 (57.1)	

* P<0.05, ** P<0.001.

^a^ Unmarried variable included single/divorced/separated/widowed.

^b^ Type of cancer: multiple responses allowed.

^c^ Type of treatment: multiple responses allowed.

^d^ Normal: 0–7 points, borderline: 8–10 points, caseness: 11–21 points

^e^ <4: no or mild distress, ≥ 4: clinically significant distress Bold values indicate statistically significant differences (P < 0.05).

Prostate cancer was the most common diagnosis among participants (41.1%), followed by lung cancer (15.5%). Regarding treatment, 52.4% of participants had undergone surgical therapy, and 46.3% had received chemotherapy. CAM utilization was significantly higher among patients diagnosed with prostate cancer (51.0%, p = 0.001) and among those who had undergone surgical treatment (56.7%, p = 0.043).

An analysis of psychological status demonstrated that CAM users reported significantly lower levels of anxiety (p = 0.016) but higher levels of depression (p = 0.014) compared to non-users. Although CAM use was more frequent among those with a distress score ≥4 (63.7%) compared with those scoring <4 (36.3%), this difference was not statistically significant.

### 3.2 Types of CAM used and reasons

The distribution of CAM modalities among elderly cancer patients who reported CAM use is presented in **Table 2**. Among CAM users, brisk walking for 1 h was the most prevalent CAM intervention, reported by 49.4% of the patients. This was followed by dietary modifications (42.9%) and the use of nutritional supplements such as vitamins (35.1%) and omega-3 fatty acids (28.2%).

**Table 2:** Types of CAM used by cancer patients (n = 245).

Used CAM modalities a	N (%)
Natural products	
Dietary (black beans, mixed grains)	105 (42.9)
Vitamins	86 (35.1)
Omega3	69 (28.2)
Ginseng, Red ginseng	55 (22.4)
Mushrooms (Chaga, Phellinus linteus)	31 (12.7)
Baked garlic	13 (5.3)
Green tea or herbal	12 (4.9)
Others	63 (25.7)
Mind and body therapy	
Brisk walking for 1 h	121 (49.4)
Prayer, meditation	10 (4.1)
Psychotherapy	5 (2.0)
Hypogastric breathing	2 (0.8)
Manipulative and body-based practices	
Hand acupuncture	7 (2.9)
Massage	6 (2.4)
Acupressure therapy	3 (1.2)
Manual therapy	1 (0.4)
Other CAM practices	
Fenbendazole	1 (0.4)
Canine meat soup	1 (0.4)

^a^ Multiple responses allowed.

^*^ Percentages do not total 100% due to multiple responses.

### 3.3 Reasons for CAM use and sources of information

The reasons for CAM utilization among elderly cancer patients are summarized in **Table 3**. The most frequently reported motivation was to strengthen immunity (61.6%), followed by improving quality of life (30.6%). Other reported reasons included the belief that CAM is natural and without side effects (26.1%), can prevent cancer recurrence (15.1%), can treat cancer itself (12.7%), and can prolong life (8.6%).

**Table 3:** Reasons for CAM use and sources of CAM information among cancer patients.

Category*	N (%)
Reasons for using CAMa	393 (96.0)
Strengthen immunity	151 (61.6)
Improve quality of life	75 (30.6)
Belief that CAM is natural; has no side effect	64 (26.1)
Prevent recurrence of cancer	37 (15.1)
To treat cancer	31 (12.7)
To live longer	21 (8.6)
Others	14 (5.6)
Sources of CAM informationa	338 (82.0)
Family or relatives	102 (42.1)
TV (news or advertisement); newspaper	69 (28.5)
Medical staff (doctors, nurses)	43 (17.8)
Internet; YouTube; social media	40 (16.5)
Neighbors or friends	39 (16.1)
Other patients	11 (4.5)
Pharmacist	5 (2.1)
Literature	5 (2.1)
Traditional Korean medicine hospitals	1 (0.4)
Others	23 (9.5)

^a^ Multiple responses allowed; therefore, percentages do not total 100%.

Sources of CAM information (n=338) varied considerably. Family or relatives were the most common source (42.1%), followed by media such as television (news or advertisements) and newspapers (28.5%). Medical staff (17.8%), Internet sources such as YouTube (16.5%), and neighbors or friends (16.1%) also contributed notably.

### 3.4 Factors influencing participants’ use of CAM

The results of the multivariate logistic regression analysis are presented in **Table 4**. Elderly cancer patients with a spouse were significantly more likely to use CAM than those without a spouse (OR = 2.493, p=0.002). A diagnosis of prostate cancer was also positively associated with CAM use (OR = 2.136, p=0.001). Similarly, patients who had undergone surgical treatment were more likely to use CAM (OR = 1.615, 95% CI = 1.065–2.449, p=0.024). In contrast, perceived anxiety, depression, and distress levels were not significantly associated with CAM use in the adjusted model.

**Table 4:** Multivariate logistic regression analysis of CAM use by demographic and clinical factors.

Category	OR	95% CI	*P*-value
Marital status			
No spouse	1	Ref.	
With spouse	2.493	1.417–4.384	0.002*
Type of cancer			
Non-prostate cancer	1	Ref.	
Prostate cancer	2.136	1.394–3.273	0.001*
Surgery treatment			
No	1	Ref.	
Yes	1.615	1.065–2.449	0.024*
Perceived anxiety			
0–7 points	1	Ref.	
Above 8 points	0.839	0.530–1.328	0.454
Perceived depression			
0–7 points	1	Ref.	
Above 8 points	0.654	0.389–1.100	0.110
Perceived distress level			
≥ 4 points	1	Ref.	
< 4 points	1.435	0.939–2.914	0.095

OR, odds ratio; CI, confidence interval; Ref., reference category.

*Statistically significant at P < 0.05.

## 4 Discussion

This study provides evidence on the prevalence and determinants of CAM use among elderly cancer patients in Korea, a population often underrepresented in supportive care research. The overall CAM utilization rate was 60%, considerably higher than previously reported rates, such as 27% among US veterans with cancer and 51% in a recent global systematic review (22, 23). However, this prevalence estimate should be interpreted with caution, as the operational definition of CAM in this study included lifestyle-based practices such as brisk walking. This broader definition may have increased the observed prevalence and may limit direct comparability with studies using narrower CAM definitions. Multivariate analysis identified marital status, surgical treatment, and prostate cancer diagnosis as significant predictors of CAM use. Notably, the high uptake of CAM among elderly Korean men contrasts with earlier findings that male patients are generally less likely to engage in CAM (24). This discrepancy may reflect sociocultural dynamics unique to Korea, including the influence of family caregiving patterns and strong cultural trust in traditional medicine. It may also partly reflect the characteristics of the study population and the broader operational definition of CAM adopted in this study. The importance of proactive clinician–patient dialogue regarding CAM, particularly with elderly male patients, underscores the need to integrate culturally sensitive supportive strategies into cancer care (25).

Our study highlights important implications for supportive cancer care in elderly patients. Exercise and dietary interventions emerged as the most practiced CAM modalities, with brisk walking reported most frequently. Brisk walking was the most frequently reported CAM modality; however, this finding should be interpreted in light of the study’s broad CAM classification. Nevertheless, lifestyle-based strategies such as exercise are feasible and generally well accepted by older patients, who often report benefits including reduced stress, improved sleep, and enhanced quality of life (26). Elderly cancer patients, in particular, perceive exercise positively, reporting improvements in physical health and emotional wellbeing, particularly in stress reduction and sleep quality (27). These findings are consistent with prior evidence demonstrating improvements in physical function, disability reduction, chronic disease prevention, and psychological health (23). Over the past 30 years, exercise oncology research has shown that higher levels of physical activity after a cancer diagnosis are associated with substantially lower mortality, with both all-cause and cancer-specific deaths reduced by over 40% in more active patients (28). In the Korean context, access to public exercise facilities and senior centers may further facilitate participation, promote social engagement, and reduce isolation (29). Collectively, these findings suggest that low-barrier, culturally appropriate lifestyle-based CAM practices may serve as supportive components of holistic, patient-centered survivorship care for elderly cancer patients; however, their effects on psychosocial wellbeing and clinical outcomes warrant cautious interpretation pending further evidence from rigorous longitudinal studies.

Depression and anxiety symptoms are highly prevalent among elderly cancer patients and significantly affect treatment adherence, clinical outcomes, and overall quality of life (30). In our study, CAM utilization appeared to be more common among patients with depressive symptoms than among those with higher anxiety scores. However, in multivariable analyses, neither depressive symptoms nor anxiety was independently associated with CAM use. These findings should therefore be interpreted with caution. Approximately two-thirds of participants reported borderline depressive symptoms, underscoring the substantial emotional burden in this population. Although this pattern is broadly consistent with previous studies suggesting that psychological distress, particularly depression, may increase interest in CAM as a means of self-management or emotional regulation, our findings do not establish depression as an independent driver of CAM use. Given that cancer-related fatigue (CRF) may further exacerbate mood disturbances, particularly depression, non-pharmacological interventions such as structured physical activity may offer benefits not only in managing fatigue but also in alleviating psychological distress within comprehensive cancer care (17).

Taken together, these findings highlight the importance of careful psychosocial assessment and open communication regarding CAM use in elderly cancer patients, while tailored exercise and dietary approaches may be considered as part of holistic, patient-centered supportive care rather than as interventions with proven effects on clinical outcomes.

Participants in this study reported using CAM primarily to boost immunity and enhance quality of life, often perceiving this intervention as safer due to their natural origin. This perception aligns with previous research showing that elderly cancer patients frequently turn to CAM to manage symptoms, improve wellbeing, and assert greater control over treatment decisions (15, 16). However, although such risks were not directly assessed in this study, prior literature has raised concerns about the potential harms associated with unsupervised CAM use in this population (31). For instance, ginseng—a commonly used CAM product—has demonstrated cytotoxic effects on certain cancer cells, yet its unregulated use may lead to unpredictable outcomes (32). Similarly, excessive consumption of dietary supplements such as vitamins can lead to complications including nephrolithiasis and hypercalcemia, thereby interfering with standard cancer therapies (33). These issues warrant careful consideration in elderly cancer care, particularly in light of concerns regarding unregulated production standards, potential herb–drug interactions, and inconsistent evidence regarding CAM efficacy (34). To mitigate these risks, healthcare providers should play a proactive role in offering balanced, evidence-based guidance on CAM use. Open and non-judgmental communication is crucial for facilitating informed and safe decision-making among elderly cancer patients (15). Furthermore, the development of trustworthy and accessible sources of CAM information is vital to support elderly cancer patients in navigating integrative care safely and effectively.

The sources of CAM information among elderly cancer patients are diverse and shaped by interpersonal and media influences. Family members, spouses, and friends were identified as the primary sources of CAM-related information, which aligns with prior studies highlighting the role of familial support in healthcare decision-making (35). Traditional media, such as television programs and newspaper articles, continue to influence while among patients’ perceptions of CAM, while digital platforms, including YouTube, are emerging as increasingly prominent, albeit limited, sources of information among older adults (20). Our findings highlight the significant role of family dynamics in facilitating CAM engagement, particularly among patients with a spouse, which is consistent with prior literature showing that families of cancer patients often express a heightened need for caregiving-related information and support (36).

Simultaneously, the proliferation of complementary and alternative medicine information through non-clinical channels has emerged as a significant challenge. The survey results of this study also reveal notable safety concerns, such as participants using remedies or preparations like “canine meat soup” and “fenbendazole” without discussing them with their physicians (37). Healthcare professionals must now urgently establish robust mechanisms to evaluate the safety, efficacy, and compatibility of such remedies with standard cancer treatments. Emerging research highlights the need for healthcare professionals to play a pivotal role by establishing clinical guidelines and evidence for CAM use, thereby helping patients navigate the growing flood of informal health advice (38). While cancer management policies are increasingly accessible online, they often fall short of meeting the nuanced needs of the elderly population (39). To bridge this gap, primary care institutions should integrate exercise therapy and structured CAM education as fundamental components of geriatric cancer treatment protocols (40). Such integration may facilitate informed decision-making through open physician–patient dialogue, reduce treatment-related risks, and support the safe and informed integration of complementary and alternative medicine into conventional cancer care.

This study has several limitations. Its cross-sectional design precludes causal inference, as the temporal relationship between CAM use and the associated factors could not be determined; therefore, reverse causation cannot be excluded. In addition, the data were collected in 2021; patterns of CAM use may have since changed due to evolving healthcare access, patient preferences, or the lasting impact of the COVID-19 pandemic. Nevertheless, CAM utilization among elderly cancer patients remains highly relevant, and our findings provide timely evidence for supportive care planning. The single-institution, predominantly male veteran sample may limit generalizability, particularly to women, younger patients, non-veteran populations, and other clinical settings, and reliance on self-reported questionnaires introduces recall bias. Furthermore, the use of convenience sampling may have introduced selection bias, and the interview-based data collection may also have increased the likelihood of social desirability bias. Moreover, details on dosage, duration, and frequency of CAM practices were not collected, and potential confounders such as socioeconomic status and comorbidities were not fully addressed. Future research should employ multicenter, longitudinal designs with updated datasets and more diverse samples and incorporate clinical endpoints to clarify the impact of CAM on adherence, survivorship, and quality of life.

## 5 Conclusion

In this study, CAM use was prevalent among elderly male cancer patients at a veterans hospital in Korea. Exercise- and diet-related practices were the most frequently reported types of CAM, and CAM use was independently associated with marital status, prostate cancer diagnosis, and surgical treatment. Considering the study’s methodological limitations, these findings should be interpreted as associative rather than causal. Further multicenter longitudinal studies are needed to confirm these results and clarify the clinical implications of CAM use in supportive cancer care.

## Data Availability

The raw data supporting the conclusions of this article will be made available by the authors, without undue reservation.
